# Constructing Equitable Health Resilience: A Call for a Systems Approach to Intersectionality

**DOI:** 10.34172/ijhpm.2023.8099

**Published:** 2023-05-21

**Authors:** AbouAli Vedadhir, Peter Bloom, Reza Majdzadeh

**Affiliations:** ^1^Faculty of Social Sciences, University of Tehran, Tehran, Iran; ^2^Department of Population Health Sciences, University of Bristol, Bristol, UK; ^3^School of Business, University of Essex, Colchester, UK; ^4^School of Health and Social Care, University of Essex, Colchester, UK

## Dear Editor,

 Resilience within the healthcare system is defined by its capacity to anticipate and respond effectively to emergencies whilst upholding its core functions.^1^ These emergencies can range from acute challenges, such as sanctions,^2^ wars, epidemics, and natural disasters like earthquakes and floods, to chronic issues like migration and drought caused by climate change.^3^ Throughout such occurrences, those exposed to death, sickness, severe injuries, discomfort (such as malnutrition) and other health problems are the society’s most vulnerable and marginalized communities. Nonetheless, not all individuals are affected equally by the shocks impinging on health, and their lived experiences in health-threatening situations differ. Some are more impacted than others due to the complex interplay between power structures and individual characteristics in the context of ‘social fields’ (in Bourdieu’s words^4,5^) such as socio-economic status or position, race, ethnicity, gender, sexual orientation, and mental and physical disabilities, resulting in social oppression and discrimination, which render them more susceptible to the harmful effects of emergencies on the greatest achievement of their life, ie, ‘health.’^6^ Indeed, comprehending how diverse types of social oppression converge to create inequalities and influence people’s health necessitates an intersectionality lens on the injustices that occur post these crises.^7^

 In a comprehensive analysis of healthcare system resilience in low- and middle-income countries, Grimm and colleagues scrutinized the priori framework for withstanding major health shocks and uncovered novel themes that demand attention to enhance health system resilience. These themes encompass strengthening trust and social capital, prioritizing leadership, governance, and coordination, and promoting community engagement.^8^ The World Health Organization (WHO) has also examined the existing frameworks in its toolkit for health system resilience post the COVID-19 pandemic and its aftermath and recommended utilizing these frameworks for operationalizing health system resilience in the form of building blocks of the WHO health system, such as governance, financing, and service delivery, which have been appended to it as a community component.^9^ However, Grimm and colleagues’ review and the WHO toolkit should have addressed what renders vulnerable individuals susceptible and how these traits should be evaluated through an intersectional lens, with interventions not stemming from this understanding.

 The COVID-19 experience has revealed that individuals from minority and marginalized groups, who may encounter social discrimination and historical and structural oppression in their everyday life, are often the most vulnerable to adverse impacts of the emergency, such as illness, job loss, modern slavery, domestic violence, and financial instability. A lack of access to healthcare services and substandard living and working conditions may compound this vulnerability. Likewise, intersecting identities affect vulnerability and resilience during the crisis. For instance, elderly and disabled individuals may be more susceptible to the adverse effects of COVID-19.^7,10,11^

 The intersectionality approach has been recognized to address equity in resilience. This approach has been used to identify groups at risk of health insecurity,^11-13^ and amplify their voices for better representation.^14^ A more systematic intervention design approach for resilience empowerment to vulnerability has been adopted to address uncertainty conditions.^15,16^ In a project aimed at building resilience to systemic issues in the health system, intersectionality has been utilized to identify problems and enhance health system leadership.^17^

 The authors of this letter confidently assert that intersectionality is the ultimate solution to address the deeply entrenched health inequalities, especially in vulnerable communities during emergencies. However, their firsthand experience reveals that the current state of the healthcare system needs to allow for the seamless integration of the intersectional approach. In order to create effective and holistic interventions, it is essential to recognize and address intersectionality as an overtly comprehensive approach to systematic injustice and the complex web of structural inequalities in healthcare systems worldwide. This requires understanding how individuals’ multiple and fluid identities intersect and interact with various power arrangements, which in turn necessitates integrated and multi-faceted responses that consider a range of different environments and contexts. Failure to acknowledge the complexity of intersectionality can result in incomplete or inadequate interventions that do not adequately address the needs of diverse communities. Fundamental changes are, therefore, necessary for all aspects of the healthcare system to elevate intersectionality from being just a research-based tool for identifying at-risk groups to a transformative instrument for designing effective health interventions and actions. Without the ability to collect, analyze, link and use data in a way that facilitates the identifying and understanding of intersectional entities or categories for vulnerabilities, integrating and institutionalizing the intersectionality in the healthcare system will remain an unattainable goal. Decision-making processes should involve diverse representation, including individuals with diverse backgrounds, experiences, and perspectives, to incorporate intersectionality into governance, finance, and service provision. This can lead to the development of targeted interventions that address the specific needs of the most marginalized groups, such as allocating resources to areas with higher levels of need or providing culturally sensitive, people-centered and relevant health services.

 Furthermore, it is crucial to embed intersectionality into the human resources aspect of healthcare by integrating it into the educational system of front-line healthcare workers.^18,19^ Theoretical and abstract training programs on resilience, justice, equality, and intersectionality should not suffice; they should be complemented with educational practicums to instil a more reflexive practice. This will empower health service providers to take and utilize an intersectional approach in being well-prepared for a time of emergency (as Andrew Lakoff accentuated in the Unprepared),^20^ identifying and tackling health inequalities during emergencies based on the social identities and fields of people and delivering appropriate and socially just and culturally safe care.

## Ethical issues

 Not applicable.

## Competing interests

 Authors declare that they have no competing interests.
